# Supplementary material for: Hot spot prediction in protein-protein interactions by an ensemble system
Source: BMC Syst Biol. 2018 Dec 31;12(Suppl 9):132. doi: 10.1186/s12918-018-0665-8 (PMC6311905; doi:10.1186/s12918-018-0665-8)
Supplement: Supplementary file 2 — All relevant MATLAB code. A simple Matlab implement of our predictor. (ZIP 19,742 kb) [file 12918_2018_665_MOESM2_ESM.zip › Additional file 2/Read ASA/ASA Property/BID/2HHBD.txt]

 NetSurfP - Protein Surface Accessibility and Secondary Structure Predictions

  

|  |  |  |
| --- | --- | --- |
|  |  | NetSurfP - Protein Surface Accessibility and Secondary Structure Predictions  Technical University of Denmark |

  


---

```
# For publication of results, please cite:
# A generic method for assignment of reliability scores applied to solvent accessibility predictions.
# Bent Petersen, Thomas Nordahl Petersen, Pernille Andersen, Morten Nielsen and Claus Lundegaard
# BMC Structural Biology 2009, 9:51 doi:10.1186/1472-6807-9-51
#
# Column 1: Class assignment - B for buried or E for Exposed - Threshold: 25% exposure, but not based on RSA
# Column 2: Amino acid
# Column 3: Sequence name
# Column 4: Amino acid number
# Column 5: Relative Surface Accessibility - RSA
# Column 6: Absolute Surface Accessibility
# Column 7: Z-fit score for RSA prediction
# Column 8: Probability for Alpha-Helix
# Column 9: Probability for Beta-strand
# Column 10: Probability for Coil
E V  2HHBD                  1    0.657 101.027  -1.244   0.003   0.003   0.994
E H  2HHBD                  2    0.619 112.687   0.825   0.018   0.088   0.893
B L  2HHBD                  3    0.145  26.604   0.317   0.005   0.045   0.951
E T  2HHBD                  4    0.422  58.490   1.523   0.018   0.019   0.964
E P  2HHBD                  5    0.671  95.172   1.151   0.970   0.001   0.030
E E  2HHBD                  6    0.697 121.679   1.752   0.923   0.002   0.076
B E  2HHBD                  7    0.078  13.557   0.374   0.923   0.002   0.076
B K  2HHBD                  8    0.211  43.444   2.048   0.970   0.001   0.030
E S  2HHBD                  9    0.588  68.890   1.800   0.970   0.001   0.030
B A  2HHBD                 10    0.187  20.596   2.201   0.988   0.000   0.012
B V  2HHBD                 11    0.015   2.229   1.782   0.988   0.000   0.012
E T  2HHBD                 12    0.383  53.108   2.360   0.988   0.000   0.012
E A  2HHBD                 13    0.547  60.257   1.747   0.970   0.001   0.030
B L  2HHBD                 14    0.060  11.041   0.477   0.970   0.001   0.030
B W  2HHBD                 15    0.072  17.412   0.765   0.923   0.002   0.076
E G  2HHBD                 16    0.624  49.132   1.870   0.782   0.003   0.216
E K  2HHBD                 17    0.492 101.184   1.642   0.600   0.003   0.397
B V  2HHBD                 18    0.089  13.618   0.133   0.339   0.016   0.645
E N  2HHBD                 19    0.596  87.196   1.012   0.053   0.005   0.942
E V  2HHBD                 20    0.419  64.354   0.432   0.246   0.004   0.750
E D  2HHBD                 21    0.706 101.720   0.558   0.181   0.016   0.803
E E  2HHBD                 22    0.594 103.737   1.383   0.622   0.015   0.363
B V  2HHBD                 23    0.114  17.599   0.645   0.879   0.010   0.111
B G  2HHBD                 24    0.031   2.463   0.202   0.970   0.001   0.030
B G  2HHBD                 25    0.234  18.408   1.192   0.970   0.001   0.030
E E  2HHBD                 26    0.372  65.058   1.696   0.988   0.000   0.012
B A  2HHBD                 27    0.031   3.471   0.950   0.988   0.000   0.012
B L  2HHBD                 28    0.030   5.401   0.812   0.988   0.000   0.012
B G  2HHBD                 29    0.242  19.022   1.071   0.988   0.000   0.012
B R  2HHBD                 30    0.242  55.441   1.110   0.988   0.000   0.012
B L  2HHBD                 31    0.036   6.628   0.652   0.970   0.001   0.030
B L  2HHBD                 32    0.057  10.418   0.761   0.970   0.001   0.030
E V  2HHBD                 33    0.434  66.660   0.688   0.858   0.002   0.139
E V  2HHBD                 34    0.337  51.858   0.442   0.717   0.014   0.269
B Y  2HHBD                 35    0.132  28.315   1.194   0.109   0.005   0.886
E P  2HHBD                 36    0.344  48.856  -0.105   0.246   0.004   0.750
E W  2HHBD                 37    0.544 130.736   0.058   0.406   0.004   0.590
B T  2HHBD                 38    0.073  10.111  -0.054   0.717   0.014   0.269
B Q  2HHBD                 39    0.243  43.346   0.856   0.751   0.050   0.199
E R  2HHBD                 40    0.587 134.446   0.536   0.717   0.014   0.269
B F  2HHBD                 41    0.198  39.819   0.633   0.717   0.014   0.269
B F  2HHBD                 42    0.043   8.530   0.043   0.430   0.016   0.555
E E  2HHBD                 43    0.539  94.093   0.689   0.406   0.004   0.590
E S  2HHBD                 44    0.505  59.139  -0.415   0.406   0.004   0.590
B F  2HHBD                 45    0.149  29.985  -0.374   0.321   0.003   0.675
E G  2HHBD                 46    0.512  40.279  -1.085   0.176   0.004   0.820
E D  2HHBD                 47    0.626  90.149  -1.175   0.058   0.017   0.925
E L  2HHBD                 48    0.304  55.626  -0.834   0.115   0.016   0.868
E S  2HHBD                 49    0.522  61.178  -1.232   0.115   0.016   0.868
E T  2HHBD                 50    0.471  65.355  -0.177   0.257   0.016   0.727
E P  2HHBD                 51    0.377  53.496  -1.176   0.430   0.016   0.555
E D  2HHBD                 52    0.637  91.835  -0.939   0.430   0.016   0.555
E A  2HHBD                 53    0.431  47.485  -0.968   0.430   0.016   0.555
E V  2HHBD                 54    0.307  47.217  -0.769   0.339   0.016   0.645
E M  2HHBD                 55    0.551 110.215  -0.874   0.181   0.016   0.803
E G  2HHBD                 56    0.600  47.259  -1.560   0.058   0.017   0.925
B N  2HHBD                 57    0.247  36.205  -0.959   0.018   0.019   0.964
E P  2HHBD                 58    0.619  87.879  -0.794   0.694   0.003   0.303
E K  2HHBD                 59    0.502 103.261   1.097   0.802   0.014   0.185
B V  2HHBD                 60    0.059   9.145   0.035   0.923   0.002   0.076
E K  2HHBD                 61    0.280  57.637   0.869   0.970   0.001   0.030
E A  2HHBD                 62    0.389  42.879   0.654   0.988   0.000   0.012
B H  2HHBD                 63    0.057  10.314   0.512   0.970   0.001   0.030
B G  2HHBD                 64    0.030   2.330  -0.829   0.988   0.000   0.012
E K  2HHBD                 65    0.268  55.086   0.667   0.988   0.000   0.012
B K  2HHBD                 66    0.244  50.191   1.256   0.988   0.000   0.012
B V  2HHBD                 67    0.048   7.393   0.728   0.988   0.000   0.012
B L  2HHBD                 68    0.055  10.071   0.304   0.988   0.000   0.012
E G  2HHBD                 69    0.289  22.776   0.614   0.988   0.000   0.012
B A  2HHBD                 70    0.089   9.830   0.214   0.988   0.000   0.012
B F  2HHBD                 71    0.073  14.671   0.310   0.988   0.000   0.012
B S  2HHBD                 72    0.164  19.279   0.121   0.988   0.000   0.012
E D  2HHBD                 73    0.371  53.504   0.874   0.988   0.000   0.012
B G  2HHBD                 74    0.067   5.273   0.148   0.970   0.001   0.030
B L  2HHBD                 75    0.038   6.958  -0.309   0.923   0.002   0.076
E A  2HHBD                 76    0.584  64.390   1.690   0.782   0.003   0.216
E H  2HHBD                 77    0.360  65.575   1.028   0.502   0.002   0.495
B L  2HHBD                 78    0.110  20.141   0.438   0.406   0.004   0.590
E D  2HHBD                 79    0.623  89.818   0.070   0.246   0.004   0.750
E N  2HHBD                 80    0.523  76.611   1.147   0.321   0.003   0.675
B L  2HHBD                 81    0.064  11.663   0.369   0.858   0.002   0.139
E K  2HHBD                 82    0.464  95.404   1.251   0.923   0.002   0.076
E G  2HHBD                 83    0.523  41.160   1.112   0.970   0.001   0.030
B T  2HHBD                 84    0.091  12.677   1.251   0.970   0.001   0.030
B F  2HHBD                 85    0.041   8.189   0.369   0.970   0.001   0.030
E A  2HHBD                 86    0.429  47.298   1.998   0.970   0.001   0.030
E T  2HHBD                 87    0.474  65.772   1.992   0.970   0.001   0.030
B L  2HHBD                 88    0.035   6.335   0.472   0.970   0.001   0.030
B S  2HHBD                 89    0.036   4.254  -0.571   0.970   0.001   0.030
E E  2HHBD                 90    0.558  97.535   1.819   0.923   0.002   0.076
E L  2HHBD                 91    0.397  72.617   2.107   0.858   0.002   0.139
B H  2HHBD                 92    0.084  15.207   0.467   0.782   0.003   0.216
B C  2HHBD                 93    0.159  22.267   0.042   0.600   0.003   0.397
E D  2HHBD                 94    0.653  94.169   0.413   0.622   0.015   0.363
E K  2HHBD                 95    0.585 120.252   0.766   0.430   0.016   0.555
E L  2HHBD                 96    0.283  51.762   0.435   0.113   0.043   0.844
E H  2HHBD                 97    0.670 121.818   0.348   0.018   0.047   0.935
B V  2HHBD                 98    0.046   7.132  -0.262   0.005   0.015   0.979
E D  2HHBD                 99    0.403  58.043   1.386   0.018   0.019   0.964
E P  2HHBD                100    0.407  57.782  -0.222   0.430   0.016   0.555
E E  2HHBD                101    0.580 101.309   0.360   0.522   0.016   0.462
B N  2HHBD                102    0.152  22.194   0.909   0.717   0.014   0.269
B F  2HHBD                103    0.040   7.968   0.702   0.938   0.007   0.055
E R  2HHBD                104    0.386  88.463   1.394   0.975   0.003   0.022
B L  2HHBD                105    0.217  39.788   1.067   0.970   0.001   0.030
B L  2HHBD                106    0.020   3.735   0.648   0.970   0.001   0.030
B G  2HHBD                107    0.127   9.979  -0.044   0.988   0.000   0.012
E N  2HHBD                108    0.409  59.922   0.600   0.988   0.000   0.012
B V  2HHBD                109    0.051   7.869   0.737   0.988   0.000   0.012
B L  2HHBD                110    0.021   3.772   1.353   0.988   0.000   0.012
B V  2HHBD                111    0.165  25.376   0.646   0.988   0.000   0.012
B C  2HHBD                112    0.250  35.114   0.759   0.988   0.000   0.012
B V  2HHBD                113    0.060   9.253   0.498   0.988   0.000   0.012
B L  2HHBD                114    0.032   5.914   0.406   0.970   0.001   0.030
E A  2HHBD                115    0.308  33.997   0.743   0.970   0.001   0.030
E H  2HHBD                116    0.653 118.781   0.941   0.923   0.002   0.076
E H  2HHBD                117    0.338  61.409   0.547   0.782   0.003   0.216
B F  2HHBD                118    0.150  30.185   0.663   0.321   0.003   0.675
E G  2HHBD                119    0.582  45.811   0.128   0.033   0.001   0.966
E K  2HHBD                120    0.763 156.867   0.543   0.053   0.005   0.942
E E  2HHBD                121    0.620 108.279  -0.106   0.109   0.005   0.886
B F  2HHBD                122    0.077  15.434   0.154   0.018   0.019   0.964
E T  2HHBD                123    0.341  47.269   1.441   0.016   0.005   0.979
E P  2HHBD                124    0.617  87.524   0.407   0.923   0.002   0.076
E P  2HHBD                125    0.510  72.397   1.186   0.923   0.002   0.076
B V  2HHBD                126    0.047   7.285   0.841   0.970   0.001   0.030
E Q  2HHBD                127    0.255  45.454   1.843   0.970   0.001   0.030
E A  2HHBD                128    0.475  52.345   1.784   0.970   0.001   0.030
B A  2HHBD                129    0.031   3.405   0.661   0.970   0.001   0.030
B Y  2HHBD                130    0.033   6.967   0.776   0.970   0.001   0.030
E Q  2HHBD                131    0.353  62.992   2.104   0.970   0.001   0.030
E K  2HHBD                132    0.377  77.528   2.045   0.988   0.000   0.012
B V  2HHBD                133    0.033   5.087   0.473   0.988   0.000   0.012
B V  2HHBD                134    0.087  13.387   1.005   0.988   0.000   0.012
E A  2HHBD                135    0.336  36.994   1.122   0.988   0.000   0.012
B G  2HHBD                136    0.194  15.260   1.537   0.970   0.001   0.030
B V  2HHBD                137    0.034   5.210   0.555   0.988   0.000   0.012
B A  2HHBD                138    0.059   6.458   0.761   0.988   0.000   0.012
E N  2HHBD                139    0.376  55.032   1.157   0.988   0.000   0.012
B A  2HHBD                140    0.120  13.235   1.351   0.988   0.000   0.012
B L  2HHBD                141    0.073  13.311   0.383   0.970   0.001   0.030
E A  2HHBD                142    0.254  28.035   0.994   0.923   0.002   0.076
E H  2HHBD                143    0.628 114.179   0.696   0.858   0.002   0.139
E K  2HHBD                144    0.487 100.258   1.100   0.600   0.003   0.397
B Y  2HHBD                145    0.278  59.302   0.231   0.321   0.003   0.675
E H  2HHBD                146    0.702 127.603  -0.052   0.003   0.003   0.994

Explain the output.  Go back.
```

---
